# Melatonin inhibits FAK signaling to suppress PD-L1 expression and enhance chemosensitivity in triple-negative breast cancer

**DOI:** 10.7150/ijms.127669

**Published:** 2026-01-30

**Authors:** Cheng-Che Wu, Ping-Fu Yang, Shu-Jyuan Chang, Mei-Ren Pan, Chung-Liang Li, Chun-Chieh Wu, Jung-Yu Kan, Fang-Ming Chen, Ming-Feng Hou, Chi-Wen Luo

**Affiliations:** 1 Division of Breast Oncology and Surgery, Department of Surgery, Kaohsiung Medical University Hospital, Kaohsiung Medical University, Kaohsiung, Taiwan.; 2 Drug Development and Value Creation Research Center, Kaohsiung Medical University, Kaohsiung, Taiwan.; 3 Graduate Institute of Clinical Medicine, Kaohsiung Medical University, Kaohsiung, Taiwan.; 4 Department of Pathology, Kaohsiung Medical University Hospital, Kaohsiung Medical University, Kaohsiung, Taiwan.; 5 Center for Liquid Biopsy and Cohort Research, Kaohsiung Medical University, Kaohsiung, Taiwan.; 6 Department of Cosmetic Science and Institute of Cosmetic Science, Chia Nan University of Pharmacy and Science, Tainan, Taiwan.

**Keywords:** Triple-negative breast cancer, melatonin, immune modulation, focal adhesion kinase (FAK), programmed death-ligand 1 (PD-L1)

## Abstract

**Background:**

Triple-negative breast cancer (TNBC) is an aggressive subtype lacking targetable hormone receptors, making conventional chemotherapy the primary treatment option, despite its associated toxicity and potential for drug resistance. Melatonin, a natural hormone with anticancer and immunomodulatory properties, has shown promise in multiple cancers; however, its role in TNBC remains unclear.

**Methods:**

We analyzed serum melatonin levels in TNBC patients and healthy controls. The biological effects of melatonin were then evaluated in human (MDA-MB-231, MDA-MB-468) and murine (4T1) TNBC cell lines. *In vitro* assays assessed proliferation, apoptosis, migration, epithelial-mesenchymal transition (EMT), and chemosensitization. Mechanistic pathways were analyzed, and an orthotopic 4T1 syngeneic mouse model was employed to confirm antitumor and immunomodulatory effects *in vivo*.

**Results:**

We found that TNBC patients had significantly lower serum melatonin levels than healthy controls. *In vitro*, melatonin reduced cell viability, migration, and tumorsphere formation, and promoted apoptosis. Mechanistically, it downregulated focal adhesion kinase (FAK) and programmed death-ligand 1 (PD-L1). FAK inhibition increased melatonin sensitivity, whereas FAK overexpression conferred resistance. Melatonin also enhanced cisplatin cytotoxicity. *In vivo*, melatonin treatment suppressed tumor growth, increased CD8⁺ T-cell infiltration, and decreased PD-L1 expression and the number of FOXP3⁺ regulatory T cells in the tumor microenvironment.

**Conclusions:**

Melatonin suppresses TNBC progression by inhibiting proliferation and migration and by modulating the immune microenvironment through the FAK-PD-L1 axis. These findings highlight melatonin as a potential low-toxicity adjunct to enhance the efficacy of current TNBC therapies.

## Introduction

Breast cancer is the most frequently diagnosed cancer and a leading cause of cancer-related mortality among women worldwide 1. Molecular classification is based on the expression of the estrogen receptor (ER), progesterone receptor (PR), and human epidermal growth factor receptor 2 (HER2) 2, 3. Triple-negative breast cancer (TNBC), defined by the absence of these markers, is a biologically unique and clinically aggressive subtype 4. Clinically, TNBC is associated with earlier metastatic spread, fewer therapeutic choices, and worse disease-specific survival compared to other subtypes 3-8. Lacking well-defined molecular targets, treatment for TNBC has largely been restricted to cytotoxic chemotherapy, which is limited by significant toxicity and modest long-term benefits 9, 10. While newer agents, including gene expression-guided drugs and immune checkpoint inhibitors (ICIs), have shown promise, their benefits are limited to selected patient subsets and resistance frequently emerges. This underscores the critical need for novel therapeutic strategies that can enhance efficacy while minimizing adverse effects.

Melatonin, a circadian-regulated indoleamine produced mainly by the pineal gland, has emerged as a promising candidate in oncology for its multifaceted biological roles 11. Beyond its function in regulating sleep, melatonin exerts potent antioxidant, anti-inflammatory, and immunomodulatory effects 12-15. Extra-pineal sources, including the gastrointestinal tract, skin, and immune cells, also contribute to its production 16. A growing body of evidence indicates that breast cancer patients may have diminished melatonin levels, potentially due to tumor-induced circadian disruption or therapeutic effects 11, 12, 17, 18. Preclinical studies have demonstrated that melatonin can inhibit tumor proliferation, migration, angiogenesis 19, and enhance sensitivity to chemotherapy and radiation by modulating key cellular pathways 20, 21. Furthermore, melatonin can potentiate conventional chemotherapeutics, mitigate their side effects, and improve patient quality of life 22. Recent studies show it can also modulate immune responses by suppressing programmed death-ligand 1 (PD-L1) expression, a critical immune checkpoint in various tumor types 23. While these anticancer actions are well-documented in hormone receptor-positive breast cancer, their relevance to TNBC has not been fully elucidated. However, the specific molecular pathways by which melatonin modulates the aggressive phenotype and the tumor immune microenvironment in TNBC remain poorly defined. Given melatonin's diverse biological functions and therapeutic potential, this study aimed to investigate its antitumor and immunomodulatory properties in TNBC. We also assessed its potential to enhance conventional chemotherapy, evaluating its promise as an adjuvant or neoadjuvant strategy for managing TNBC.

## Materials and Methods

### Cell Lines and Culture Conditions

Human TNBC cell lines (MDA-MB-231, MDA-MB-468) and the murine 4T1 line were obtained from the American Type Culture Collection (ATCC). For routine maintenance, the culture medium consisted of Dulbecco's Modified Eagle Medium (DMEM) supplemented with 10% fetal bovine serum and standard antibiotics. All cells were maintained at 37°C in a humidified 5% CO₂ incubator.

### Antibodies and Chemical Reagents

Primary antibodies targeting focal adhesion kinase (FAK), phospho-FAK (Y397), cleaved PARP, caspase-3, vimentin, β-catenin, GAPDH, PD-L1, and E-cadherin were purchased from Cell Signaling Technology (Danvers, MA, USA). The anti-mouse PD-L1 antibody (A23922) was obtained from ABclonal (Woburn, MA, USA), and horseradish peroxidase (HRP)-conjugated secondary antibodies were sourced from GeneTex (Irvine, CA, USA). The chemotherapeutic agent cisplatin and the specific FAK inhibitor PF-562271 were procured from Sigma-Aldrich (St. Louis, MO, USA) and Selleckchem (Houston, TX, USA), respectively.

### Quantification of Serum Melatonin

Circulating melatonin concentrations in serum samples were determined using a commercial enzyme-linked immunosorbent assay (ELISA) kit (MBS263138; MyBioSource, USA) according to the manufacturer's instructions. To minimize circadian variation, all blood samples were collected between 8:00 AM and 12:00 PM.

### Western Blotting

For protein analysis, cells were lysed in M-PER™ buffer, and the resulting lysates were cleared by centrifugation. Protein content was determined using a BCA assay. An equal quantity of protein from each sample was resolved by SDS-PAGE and subsequently transferred to nitrocellulose membranes. Following a blocking step, the membranes were probed with specified primary antibodies and corresponding HRP-conjugated secondary antibodies. Immunoreactive bands were visualized using an ECL detection system, as previously described 8.

### Gene Silencing and Overexpression

FAK expression was silenced by transfecting cells with a PTK2-targeting small interfering RNA (siRNA; M-003164-02-0005; Dharmacon, Lafayette, CO, USA); a non-targeting siRNA (CN-001000-01-05) was used as a negative control. For transient overexpression, cells were transfected with a human FAK expression plasmid (#186141; Addgene, Watertown, MA, USA). All transfections were performed using Lipofectamine 2000 (Thermo Fisher Scientific, Waltham, MA, USA) or X-tremeGENE^TM^ (Roche, Mannheim, Germany) transfection reagents in Opti-MEM medium with either 100 nM siRNA or the plasmid DNA.

### Quantitative Real-Time PCR (qPCR)

Total RNA was extracted from cells using TRIzol reagent. Complementary DNA (cDNA) was then synthesized from the RNA template via reverse transcription with SuperScript III. The qPCR analysis was performed on a LightCycler 480 system using primers specific for PD-L1 (F: 5′-TATGGTGGTGCCGACTACAA-3′; R: 5′-TGGCTCCCAGAATTACCAAG-3′). GAPDH (F: 5′-AAGGCTGGGGCTCATTTGC-3′; R: 5′-GCTGATGATCTTGAGGCT-3′) was used as the internal normalization control.

### Assessment of Cell Proliferation

Cells were plated in 24-well plates at a density of 1 × 10⁴ cells per well. Following a 72-hour treatment with the indicated compounds, MTT reagent was added to each well. The resulting formazan crystals were dissolved in DMSO, and the absorbance at 560 nm was measured to determine cell viability.

### Migration and Invasion Assays

Cell migration was assessed via a wound healing assay using silicone culture inserts (ibidi, Germany). Following insert removal to generate a cell-free gap, wound closure was monitored and imaged at 0 and 24 hours. For invasion analysis, a Transwell system with Matrigel-coated inserts was employed. Cells suspended in serum-free medium were placed in the upper chamber, while the lower chamber contained medium with 10% FBS as a chemoattractant. After 24-48 h, cells that had invaded through the Matrigel were fixed, stained, and counted, as described previously 8.

### Colony Formation

To evaluate long-term proliferative capacity, cells were seeded in 6-well plates and subsequently treated with melatonin at various concentrations (0 to 1.0 mM). The cells were incubated for 10-14 days to allow for colony growth. Finally, the colonies were fixed, stained with Giemsa, and counted, with results expressed relative to the control group.

### *In vivo* Tumor Model

An orthotopic tumor model was established using eight-week-old female BALB/c mice from the National Laboratory Animal Center (Taipei, Taiwan). A suspension of 2 × 10⁴ 4T1 cells in Matrigel was injected into the mammary fat pad of each mouse. Tumor growth was tracked by measuring tumor dimensions, and tumor volume was calculated using the formula: (length × width²)/2. When tumors reached approximately 100 mm³, mice were randomized into two groups (n = 5 per group). The treatment group received intraperitoneal injections of melatonin (50 mg/kg), while the control group received a vehicle solution (DMSO/PBS/Cremophor), with treatments administered five times per week. Upon completion of the study, tumor tissues were harvested for further analysis.

### Immunohistochemistry (IHC)

Harvested tumor tissues were preserved in formalin and embedded in paraffin for IHC analysis. Tissue sections underwent deparaffinization, rehydration, antigen retrieval, and blocking of endogenous peroxidase activity. The sections were then incubated overnight with primary antibodies. Signal detection was performed using the EnVision system (Dako, Denmark), and nuclei were counterstained with hematoxylin.

### Statistical Analysis

All statistical analyses were performed using SPSS software (v19.0). A two-tailed Student's t-test was used to compare differences between two experimental groups at single endpoints. Tumor growth curves were analyzed using two-way ANOVA with repeated measures. All results were considered statistically significant if the p-value was less than 0.05.

## Results

### Serum Melatonin Levels Are Significantly Reduced in Patients with TNBC

**Figure 1:**
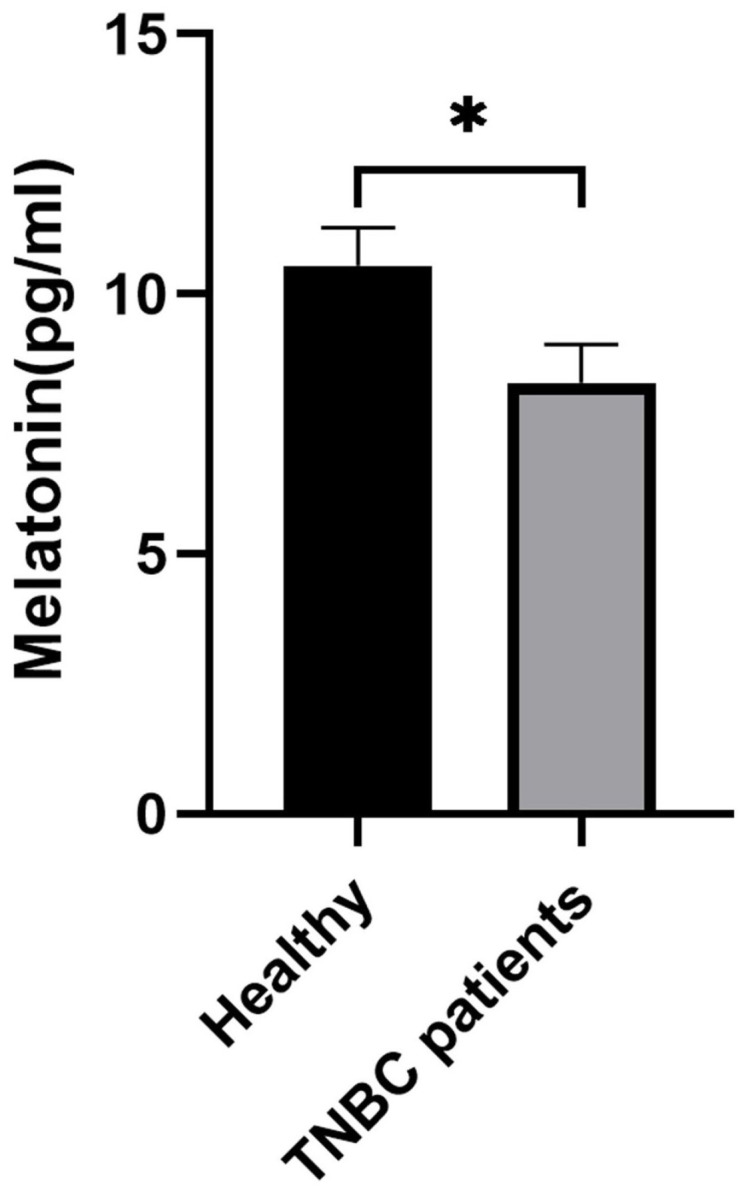
** Serum melatonin levels are significantly decreased in patients with TNBC.** Serum melatonin concentrations were measured in healthy individuals (n = 10) and patients with TNBC (n = 25) using a commercial ELISA kit. Compared with healthy controls, patients with TNBC showed a significant reduction in circulating melatonin levels (*P < 0.05). Data are presented as mean ± SEM.

To establish a potential clinical link between melatonin and TNBC, we assessed serum melatonin concentrations in healthy individuals (n = 10) and patients with TNBC (n = 25). An ELISA revealed that patients with TNBC exhibited significantly lower circulating melatonin levels compared to the healthy control group (Figure 1), suggesting an association between melatonin deficiency and TNBC pathogenesis.

### Melatonin Inhibits Cell Viability and Induces Apoptosis in TNBC Cells

**Figure 2:**
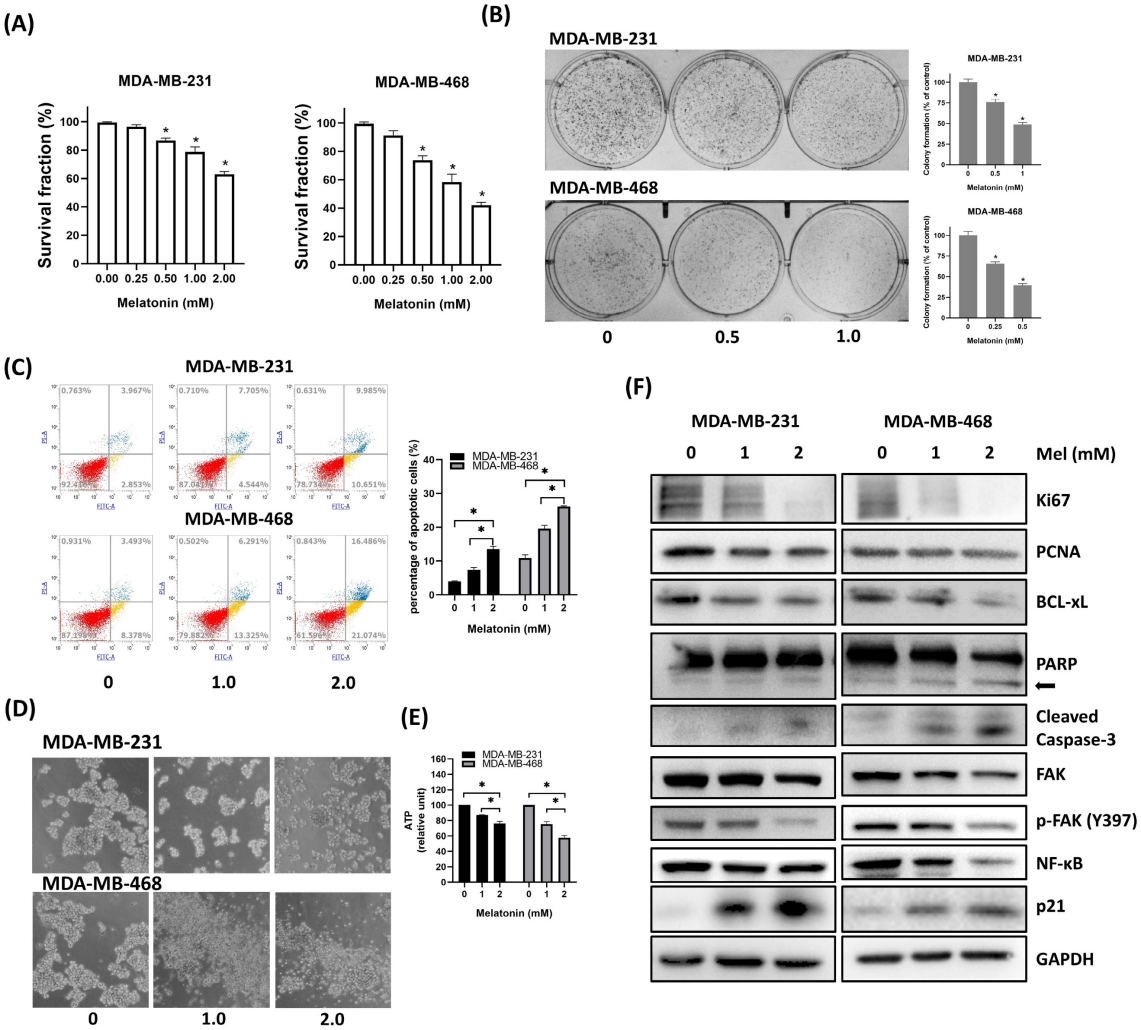
** Melatonin suppresses cell viability, disrupts tumorsphere formation, and induces apoptosis in TNBC cells. (A)** Cell viability of MDA-MB-231 and MDA-MB-468 cells was assessed by MTT assay after a 72-hour treatment with increasing concentrations of melatonin (0-2.0 mM). **(B)** Long-term clonogenic survival was assessed by colony formation assay following melatonin treatment (0, 0.5, and 1.0 mM). **(C)** Apoptosis was analyzed by flow cytometry after Annexin V/PI staining, showing a dose-dependent increase in apoptotic cells at indicated concentrations (0, 1.0, and 2.0 mM). **(D)** Representative images from tumorsphere formation assays show that melatonin disrupts 3D sphere morphology in both cell lines.** (E)** Intracellular ATP levels in tumorspheres treated with melatonin were quantified to assess metabolic activity.** (F)** Western blot analysis of indicated proteins after melatonin treatment. Melatonin decreased proliferation markers (Ki67, PCNA), anti-apoptotic BCL-xL, FAK, p-FAK (Y397), and NF-κB, while increasing p21 and apoptotic markers (cleaved PARP (arrow) and cleaved caspase-3). GAPDH served as a loading control. Data are presented as mean ± SEM from three independent experiments. *P < 0.05.

To evaluate its antitumor effects, human TNBC cell lines (MDA-MB-231 and MDA-MB-468) were treated with increasing concentrations of melatonin. This treatment resulted in a dose-dependent reduction in cell viability, as measured by MTT assays (Figure 2A), and a significant suppression of clonogenic potential in colony formation assays (Figure 2B). Consistent with these findings, flow cytometric analysis confirmed a marked increase in both early and late apoptotic cell populations following melatonin exposure (Figure 2C). In 3D tumorsphere cultures, melatonin impaired both sphere formation and structural integrity (Figure 2D), which was accompanied by a significant decrease in intracellular ATP levels, indicating compromised metabolic activity (Figure 2E). At the molecular level, Western blot analysis demonstrated that melatonin dose-dependently downregulated markers of proliferation (Ki67, PCNA) and survival (BCL-xL), as well as total and phosphorylated focal adhesion kinase (p-FAK). Notably, melatonin also induced the upregulation of p21, suggesting that cell cycle arrest contributes to its growth-suppressive effects. Consistent with the induction of apoptosis, the levels of cleaved PARP and cleaved caspase-3 were elevated, confirming the activation of apoptotic pathways. Furthermore, the expression of NF-κB was substantially downregulated, indicating the inhibition of key survival and inflammatory signaling cascades (Figure 2F). Collectively, these data show that melatonin suppresses TNBC cell proliferation and metabolic activity while promoting apoptosis by altering critical signaling pathways.

### Melatonin Suppresses TNBC Cell Migration and Reverses Epithelial-mesenchymal transition (EMT)

**Figure 3:**
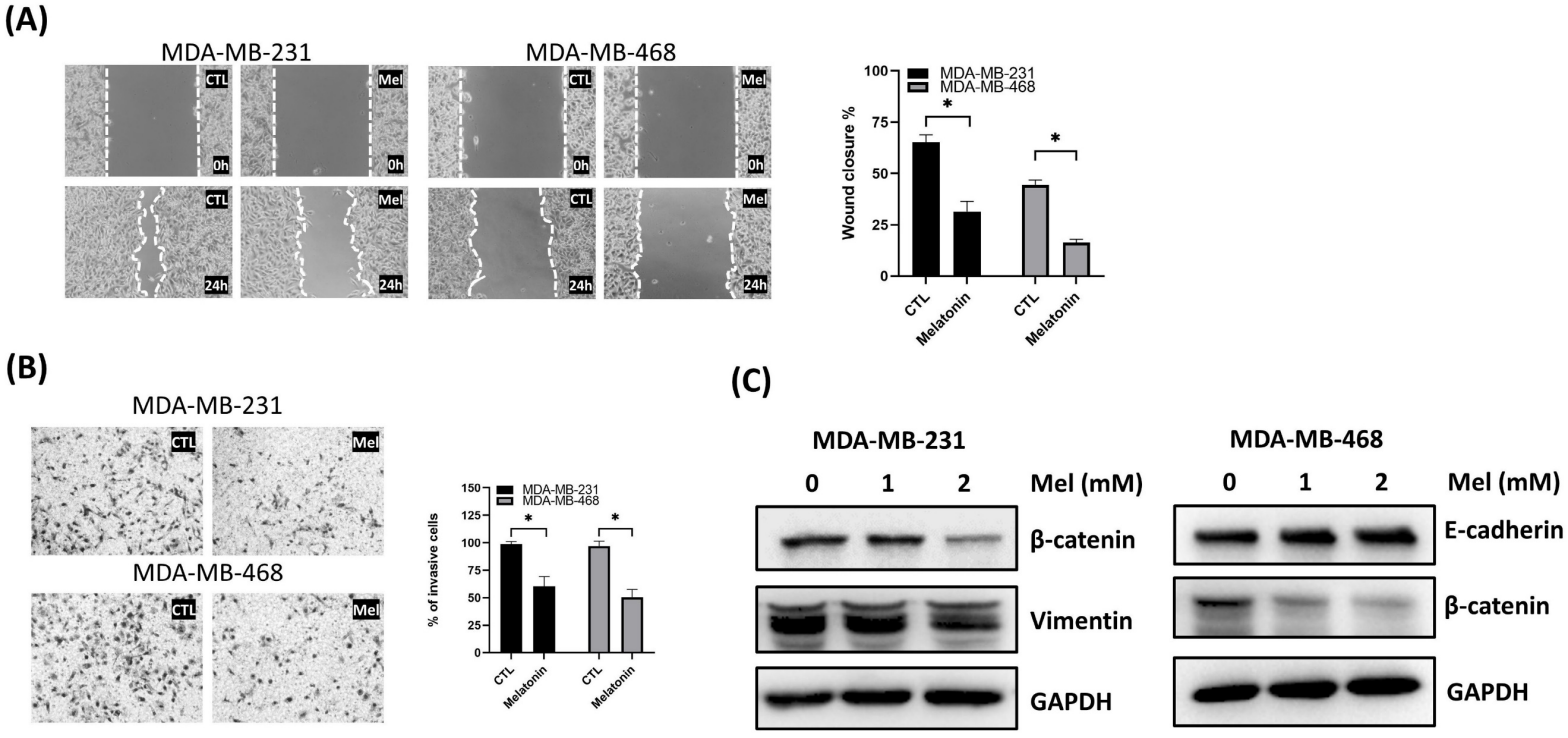
** Melatonin suppresses migration and invasion and modulates EMT-associated markers in TNBC cells. (A)** Cell migration was assessed by a wound-healing assay. Representative images (left) and quantification of wound closure (right) show that melatonin treatment for 24 h significantly reduced migration in MDA-MB-231 (2 mM) and MDA-MB-468 (0.5 mM) cells. **(B)** Cell invasion was assessed using a Transwell invasion assay. Representative images (left) and quantification (right) show a significant decrease in invasive cells following melatonin treatment at the same concentrations used in the migration assay. **(C)** Western blot analysis of EMT-associated markers after melatonin treatment (0, 1, and 2 mM; 48 h). In MDA-MB-231 cells, melatonin decreased vimentin and β-catenin levels, whereas in MDA-MB-468 cells, melatonin increased E-cadherin and decreased β-catenin. GAPDH served as a loading control. Data are presented as mean ± SEM from three independent experiments. *P < 0.05.

Having established melatonin's effects on cell viability and apoptosis, we next sought to determine its impact on TNBC cell motility and invasive potential. The effects of melatonin on the migratory and invasive behavior of TNBC cells were examined using wound healing and Transwell assays. In both MDA-MB-231 and MDA-MB-468 cells, melatonin treatment significantly inhibited wound closure over a 24-hour period (Figure 3A). Similarly, Transwell assays revealed a marked decrease in the number of invading cells in melatonin-treated groups, confirming its ability to suppress invasive capacity (Figure 3B).

To investigate the underlying molecular mechanisms, we assessed the expression of key EMT markers. Western blot analysis revealed that melatonin treatment upregulated the epithelial marker E-cadherin in MDA-MB-468 cells, while downregulating the mesenchymal marker β-catenin in both cell lines and vimentin in MDA-MB-231 cells (Figure 3C). These results indicate that melatonin impairs TNBC cell motility and invasiveness, at least in part, by reversing the EMT process, supporting its potential as an anti-metastatic agent.

### FAK Signaling Mediates the Anti-TNBC Effects of Melatonin

**Figure 4:**
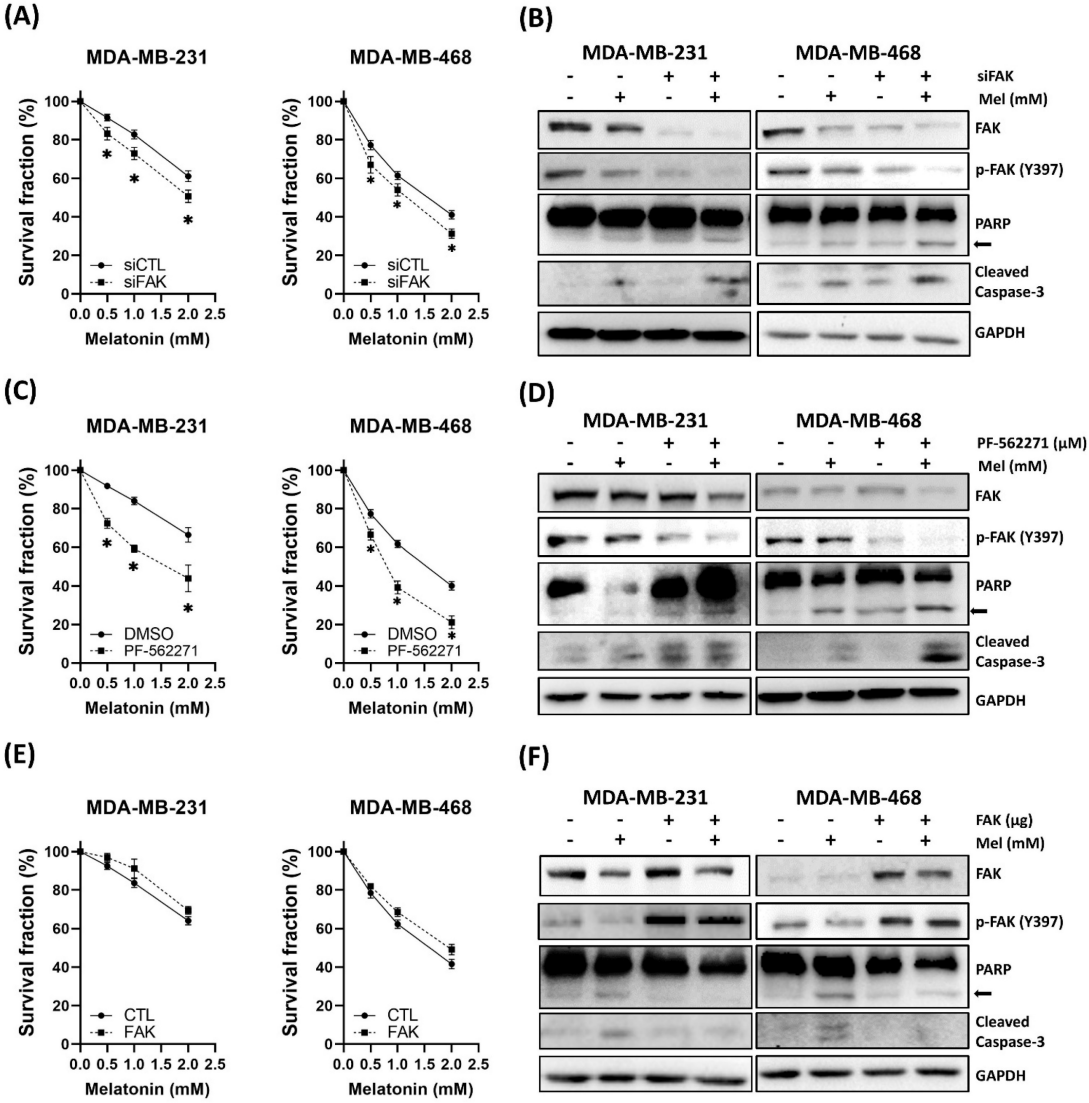
** FAK signaling modulates TNBC cell sensitivity to melatonin-induced cytotoxicity**. **(A)** MDA-MB-231 and MDA-MB-468 cells were transfected with control siRNA (siCTL) or siFAK and treated with increasing concentrations of melatonin (0-2 mM) for 72 h. Cell survival fraction was assessed by MTT assay.** (B)** Immunoblot analysis confirms FAK knockdown and shows enhanced apoptotic signaling in siFAK cells treated with melatonin (2 mM), as indicated by PARP cleavage (arrow) and cleaved caspase-3; p-FAK (Y397) is also shown.** (C)** Cells were co-treated with PF-562271 (1 μM) and melatonin (0-2 mM) for 72 h, and survival fraction was measured by MTT assay. **(D)** Western blotting shows increased apoptotic signaling (PARP cleavage (arrow) and cleaved caspase-3) in the combination treatment group (1 μM PF-562271 and 2 mM melatonin); p-FAK (Y397) is also shown. **(E)** Cells transfected with control vector (CTL) or FAK expression plasmid were treated with melatonin (0-2 mM) for 72 h, and survival fraction was measured by MTT assay. **(F)** Immunoblotting confirms increased FAK expression and shows attenuated apoptotic signaling (PARP cleavage (arrow) and cleaved caspase-3) upon melatonin treatment (2 mM); p-FAK (Y397) is also shown. GAPDH served as a loading control. Data are presented as mean ± SEM from three independent experiments. *P < 0.05.

Based on our observation that melatonin downregulated FAK (Figure 2F) and the differential sensitivity between cell lines (Figure 2A), we hypothesized that FAK signaling plays a critical role in modulating TNBC cell sensitivity to melatonin. To test this hypothesis, we modulated FAK activity through genetic and pharmacological approaches. Silencing FAK expression using siRNA enhanced the antitumor effects of melatonin, leading to further reductions in cell viability and increased apoptosis (Figure 4A-B). Similarly, pharmacological inhibition of FAK with PF-562271 potentiated melatonin-induced cytotoxicity (Figure 4C-D). Conversely, ectopic overexpression of FAK partially rescued cells from melatonin-induced cell death (Figure 4E-F). Together, these findings establish that FAK signaling is a key determinant of TNBC cell responsiveness to melatonin.

### Melatonin Downregulates PD-L1 Expression via FAK Suppression

**Figure 5:**
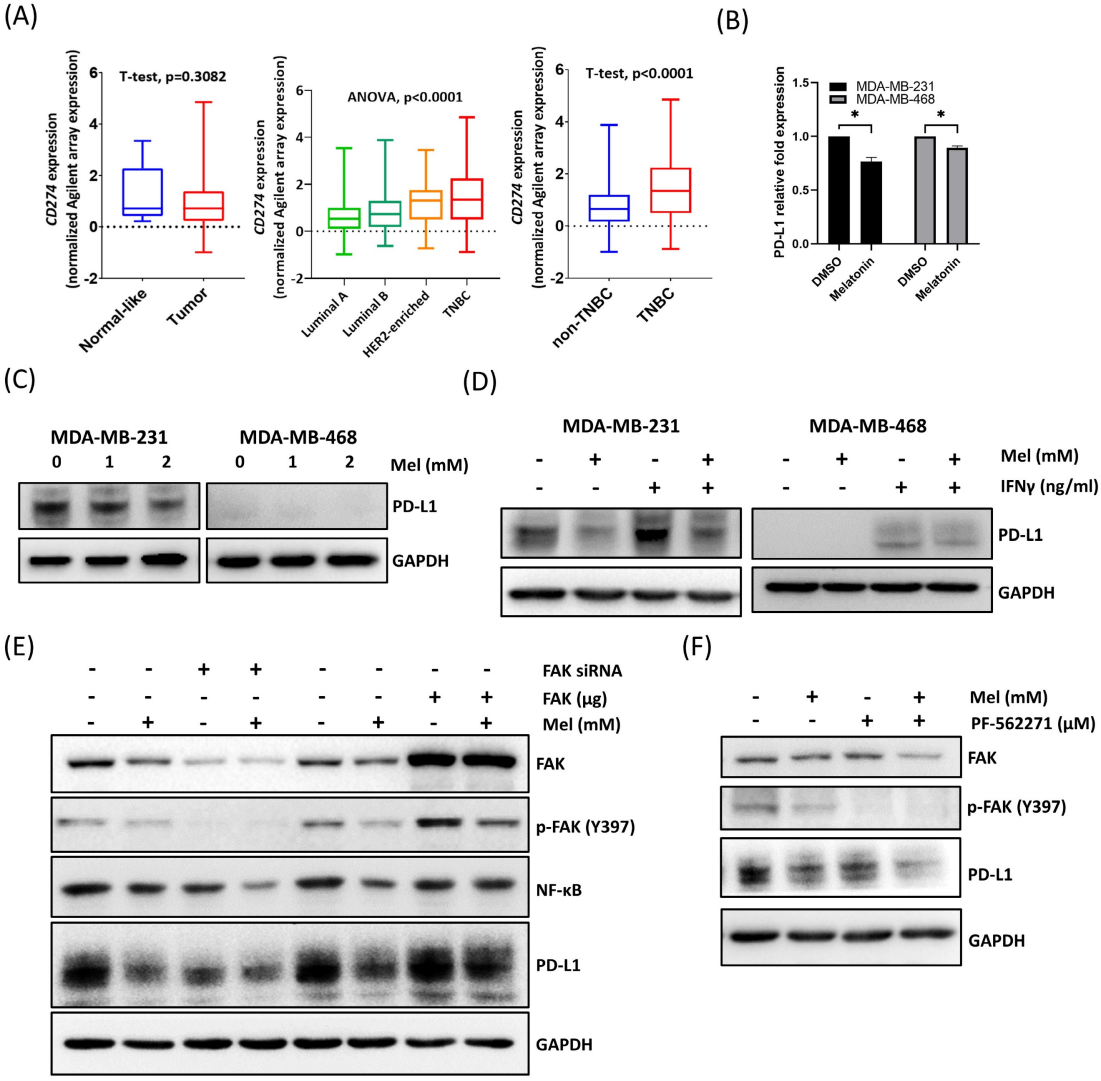
** Melatonin downregulates PD-L1 expression in TNBC cells through inhibition of the FAK signaling pathway. (A)** CD274 (PD-L1) mRNA expression analysis in the TCGA-BRCA cohort. Left: comparison between Normal-like (adjacent normal) and tumor tissues. Middle: comparison across breast cancer subtypes (Luminal A, Luminal B, HER2-enriched, and TNBC). Right: comparison between non-TNBC and TNBC, showing elevated CD274 expression in TNBC. Normal-like refers to adjacent normal tissues from breast cancer patients. Statistical tests and *P* values are indicated in the plots. **(B)** qPCR analysis showing that melatonin treatment (2 mM) significantly reduced PD-L1 (CD274) mRNA levels in MDA-MB-231 and MDA-MB-468 cells. Expression was normalized to GAPDH and presented as relative fold change.** (C)** Western blot analysis demonstrating a dose-dependent decrease in PD-L1 protein expression in cells treated with melatonin (0, 1, and 2 mM) for 48 h.** (D)** Melatonin (2 mM) attenuated IFN-γ-induced PD-L1 upregulation in TNBC cells (IFN-γ, 50 ng/mL for 48 h). **(E)** FAK silencing (FAK siRNA) enhanced the downregulation of NF-κB and PD-L1 by melatonin (2 mM), whereas FAK overexpression partially reversed these suppressive effects. **(F)** Pharmacological inhibition of FAK using PF-562271 (1 μM) further augmented the suppressive effect of melatonin (2 mM) on PD-L1 expression. GAPDH served as a loading control. Data in (B) are presented as mean ± SEM from three independent experiments. *P < 0.05.

The PD-L1/PD-1 axis plays a central role in regulating antitumor immunity 24. To confirm the clinical relevance of PD-L1 in TNBC, we analyzed the TCGA-BRCA dataset. While PD-L1 (CD274) expression did not differ significantly between tumor and adjacent normal (normal-like) tissues across the entire breast cancer cohort (Figure 5A, left), its levels were significantly higher in TNBC compared to other breast cancer subtypes (Figure 5A, middle and right). This distinct expression profile suggests that the PD-L1 axis is a particularly critical mechanism of immune evasion and a prime therapeutic target in TNBC.

Although melatonin has been reported to downregulate PD-L1 in multiple tumor types 24, evidence regarding its role in TNBC remains limited. Therefore, we investigated whether melatonin regulates PD-L1 expression in TNBC cells. Our results showed that melatonin treatment significantly reduced PD-L1 expression at both the mRNA and protein levels in MDA-MB-231 and MDA-MB-468 cells (Figure 5B, 5C). Moreover, melatonin attenuated IFN-γ-induced PD-L1 upregulation, demonstrating its ability to counteract cytokine-driven immune signaling (Figure 5D).

At the signaling level, melatonin treatment was accompanied by reduced FAK/p-FAK and NF-κB levels (Figures 2F) together with decreased PD-L1 expression (Figures 5E). Given that FAK is known to regulate immune evasion pathways, including PD-L1 expression 25-29, we hypothesized that melatonin modulates PD-L1 through the FAK signaling axis. To verify this, we performed gain- and loss-of-function analyses. As shown in Figure 5E, FAK silencing enhanced melatonin-induced PD-L1 downregulation, whereas FAK overexpression partially reversed the suppressive effect of melatonin. Notably, under these conditions, NF-κB levels changed in parallel with PD-L1 in response to FAK manipulation. Consistently, pharmacological inhibition of FAK further augmented the ability of melatonin to suppress PD-L1 expression (Figure 5F). Collectively, these results indicate that melatonin downregulates PD-L1 expression in TNBC cells primarily through the suppression of FAK signaling, accompanied by a concurrent reduction in NF-κB levels.

### Melatonin Enhances the Chemosensitivity of TNBC Cells to Cisplatin

**Figure 6:**
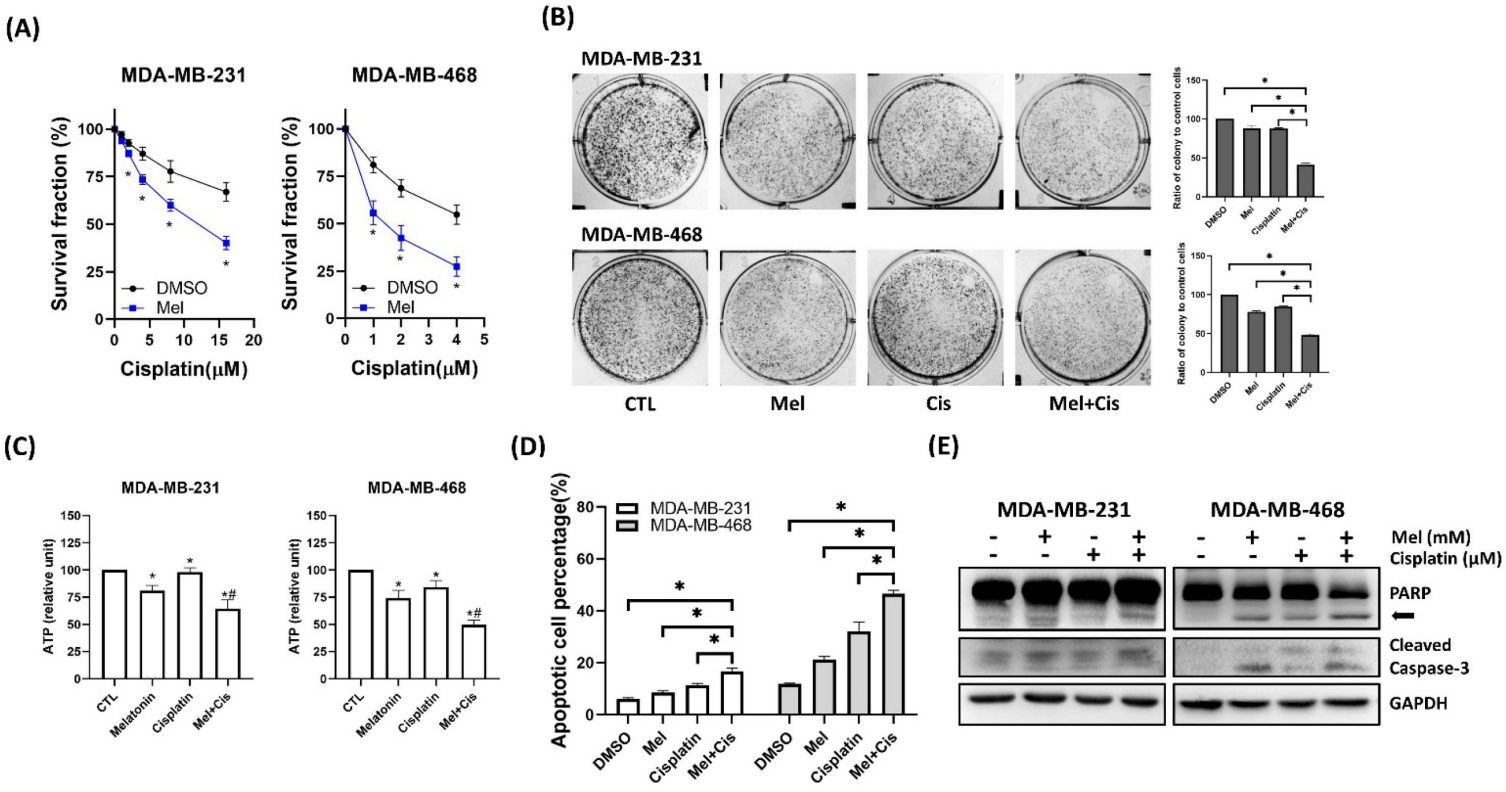
** Melatonin potentiates cisplatin-induced cytotoxicity in TNBC cells. (A)** MDA-MB-231 and MDA-MB-468 cells were co-treated with melatonin (2 mM) and increasing concentrations of cisplatin (as indicated) for 72 h, and cell survival fraction was assessed by MTT assay. **(B)** Clonogenic survival was evaluated by colony formation assay following co-treatment with melatonin (0.5 mM) and cisplatin (MDA-MB-231: 8 μM; MDA-MB-468: 4 μM). Representative images (left) and quantification (right) are shown. **(C)** Intracellular ATP levels were measured after 72 h of treatment (MDA-MB-231: 1 mM Mel + 8 μM Cis; MDA-MB-468: 0.5 mM Mel + 4 μM Cis), showing reduced metabolic activity in the combination group compared with either agent alone. **(D)** Apoptosis was quantified by flow cytometry after Annexin V/PI staining following 24 h of treatment (MDA-MB-231: 2 mM Mel + 8 μM Cis; MDA-MB-468: 1 mM Mel + 4 μM Cis), demonstrating that combined melatonin and cisplatin significantly increased the percentage of apoptotic cells relative to single treatments. **(E)** Immunoblot analysis after 48 h of treatment (MDA-MB-231: 2 mM Mel + 8 μM Cis; MDA-MB-468: 1 mM Mel + 4 μM Cis) confirms enhanced apoptotic signaling in co-treated cells, as indicated by PARP cleavage (arrow) and cleaved caspase-3. GAPDH served as a loading control. Data are presented as mean ± SEM from three independent experiments. *P < 0.05; #: *P* < 0.05 vs. melatonin or cisplatin alone.

To assess whether melatonin could potentiate conventional chemotherapy, TNBC cells were treated with cisplatin, either alone or in combination with melatonin. Co-treatment resulted in a significantly greater, dose-dependent reduction in cell viability (Figure 6A) and clonogenic potential (Figure 6B) compared to cisplatin alone. This enhanced cytotoxicity was associated with significantly decreased ATP levels in tumorspheres (Figure 6C) and increased apoptosis, as confirmed by flow cytometry (Figure 6D) and elevated levels of cleaved caspase-3 and cleaved PARP via Western blot (Figure 6E). These data demonstrate that melatonin enhances the cytotoxic efficacy of cisplatin in TNBC cells.

### Melatonin Exerts Broad Antitumor Effects in Murine 4T1 TNBC Cells

**Figure 7:**
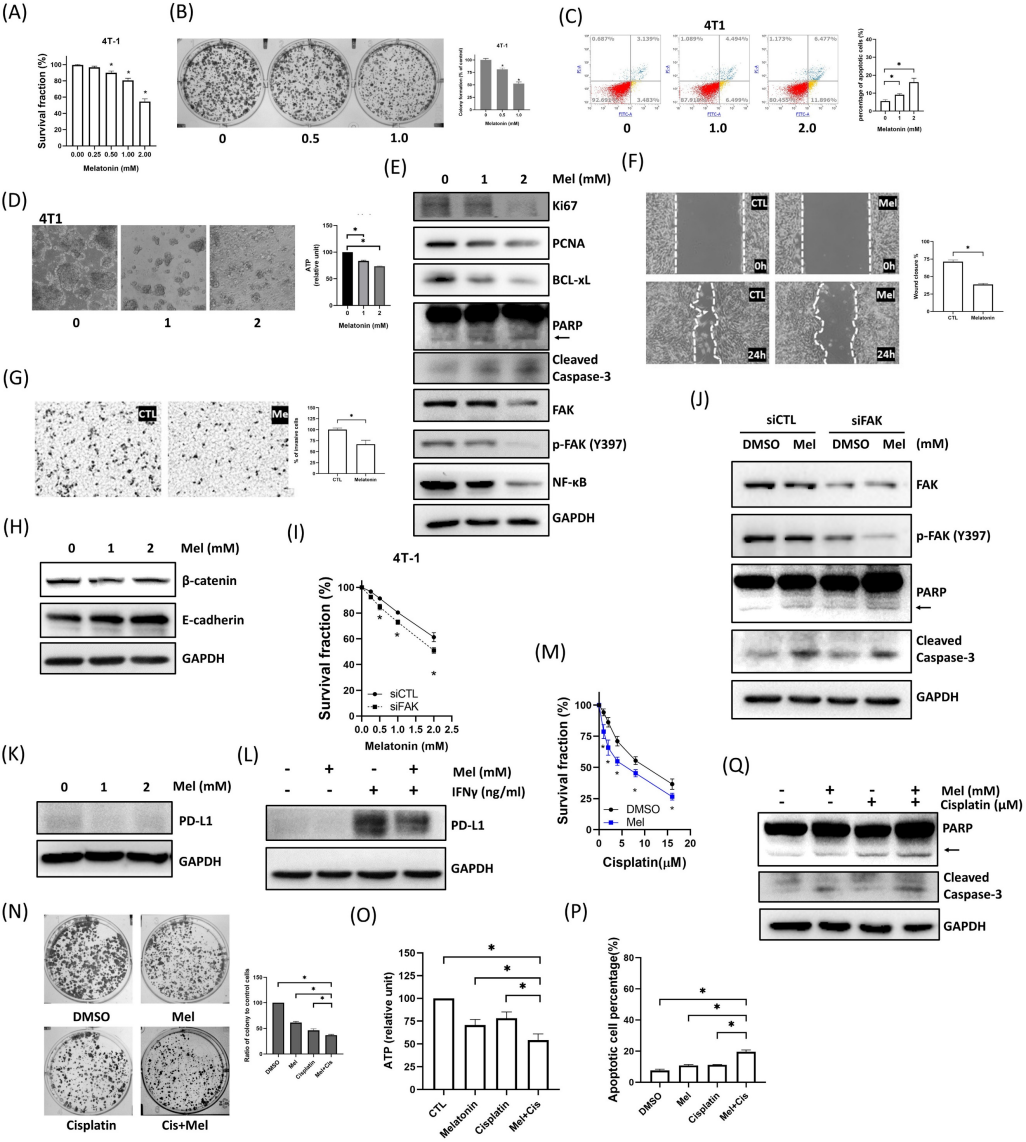
** Melatonin suppresses malignant phenotypes of murine 4T1 TNBC cells and enhances sensitivity to cisplatin. (A-E)** Melatonin reduces cell viability and induces apoptosis. 4T1 cells were treated with increasing concentrations of melatonin (0-2 mM). Melatonin dose-dependently decreased cell survival fraction **(A)** and clonogenic survival (0, 0.5, and 1.0 mM) **(B)**. Apoptosis was increased after 24 h of melatonin treatment (0, 1, and 2 mM), as assessed by Annexin V/PI flow cytometry** (C)**. Melatonin (1 and 2 mM) also disrupted tumorsphere morphology and reduced intracellular ATP levels **(D)**. Immunoblotting performed after 48 h of treatment (0-2 mM)** (E)** showed decreased proliferation markers (Ki67, PCNA) and reduced FAK pathway signaling (FAK and p-FAK (Y397)) and NF-κB, accompanied by reduced BCL-xL and increased apoptotic markers (PARP cleavage (arrow) and cleaved caspase-3). **(F-H)** Melatonin inhibits motility and modulates EMT-associated markers. Wound-healing **(F)** and Transwell invasion **(G)** assays demonstrated that melatonin (1 mM) significantly suppressed migration and invasion of 4T1 cells. Consistently, melatonin (1 and 2 mM) increased E-cadherin and decreased β-catenin** (H)**. **(I-L)** FAK mediates melatonin responses and is associated with PD-L1 regulation. FAK knockdown enhanced melatonin-induced cytotoxicity **(I)** and apoptotic signaling** (J)**. Melatonin (2 mM) reduced basal PD-L1 expression **(K)** and attenuated IFN-γ-induced PD-L1 upregulation (Melatonin: 2 mM; IFN-γ: 50 ng/mL)** (L)**. **(M-Q)** Melatonin potentiates cisplatin-induced cytotoxicity. Co-treatment with melatonin **(2 mM)** and cisplatin (increasing concentrations for **(M)**; 10 μM for **(N)**-**(Q)**) decreased survival fraction **(M)**, colony formation **(N)**, and intracellular ATP levels **(O)**, and increased apoptosis **(P)**, which was further supported by enhanced apoptotic signaling (PARP cleavage (arrow) and cleaved caspase-3) by immunoblotting **(Q)**. Data are presented as mean ± SEM from three independent experiments. *P < 0.05.

To validate our findings in a system suitable for *in vivo* studies, we confirmed that murine 4T1 TNBC cells respond to melatonin in a manner consistent with human cell lines. Indeed, melatonin treatment in 4T1 cells mirrored the effects observed in human cell lines, causing a dose-dependent reduction in viability (Figure 7A) and colony formation (Figure 7B), inducing apoptosis (Figure 7C), and impairing tumorsphere integrity and metabolic activity (Figure 7D). This was accompanied by consistent molecular changes, including the modulation of key signaling proteins (FAK, NF-κB) and apoptosis/proliferation markers (Figure 7E). Furthermore, melatonin effectively suppressed 4T1 cell migration and invasion (Figure 7F-G) and reversed EMT markers (Figure 7H). The central role of FAK was recapitulated in the 4T1 model, as FAK knockdown enhanced melatonin-induced cytotoxicity (Figure 7I-J). Similarly, melatonin suppressed both basal and IFN-γ-induced PD-L1 expression (Figure 7K-L) and effectively potentiated cisplatin to reduce viability, colony formation, and ATP levels, alongside enhanced apoptosis (Figure 7M-Q). These results confirm that melatonin inhibits multiple malignant features in 4T1 cells, consistent with our human TNBC cell models.

### Melatonin Suppresses *In vivo* Tumor Growth and Modulates the Tumor Immune Microenvironment

**Figure 8:**
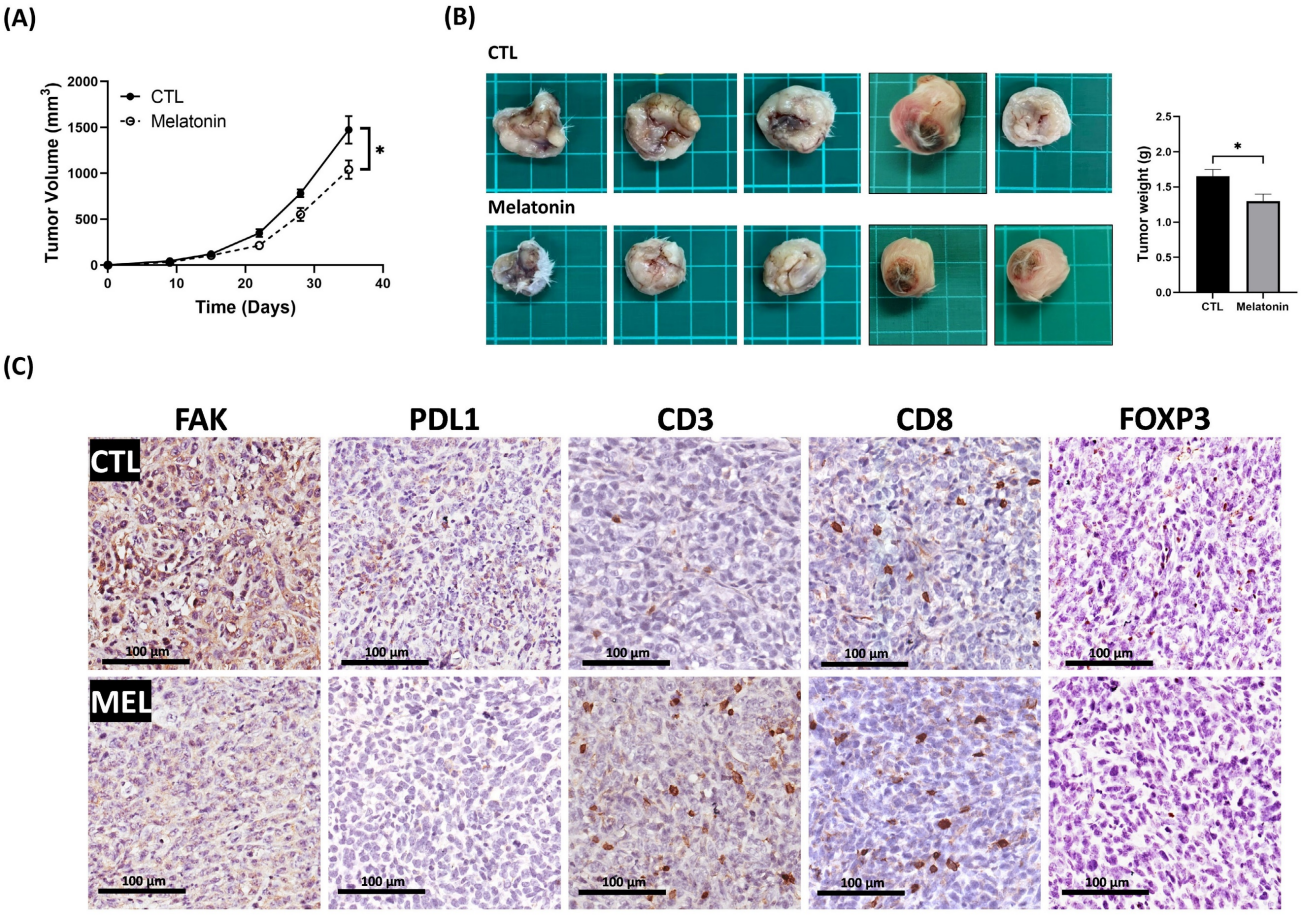
** Melatonin inhibits tumor growth and remodels the tumor immune microenvironment in a syngeneic TNBC mouse model. (A)** Tumor growth curves show that melatonin (50 mg/kg/day, five days per week; i.p.) suppressed the growth of orthotopic 4T1 tumors over the 35-day study period. **(B)** At the study endpoint (day 35), tumors were excised. Representative gross tumor images (left) and quantification of tumor weight (right) show a significant reduction in tumor burden in the melatonin-treated group compared with the CTL group. **(C)** Representative IHC images of tumor sections shows reduced FAK and PD-L1 expression in melatonin-treated tumors. Melatonin also increased infiltration of CD3⁺ and CD8⁺ T cells and decreased FOXP3⁺ regulatory T cells. Scale bars = 100 µm. Data in **(A)** and **(B)** are presented as mean ± SEM (n = 5 mice per group). *P < 0.05.

To investigate the *in vivo* efficacy of melatonin, we employed a syngeneic orthotopic 4T1 TNBC mouse model. Systemic administration of melatonin significantly reduced both tumor volume and final tumor weight compared to the vehicle-control group (p < 0.05) (Figures 8A, 8B). IHC analysis of tumor sections from melatonin-treated mice revealed a marked decrease in FAK and PD-L1 expression, consistent with our *in vitro* data. Moreover, melatonin treatment reshaped the tumor immune microenvironment, increasing CD3⁺ and CD8⁺ T-cell infiltration while reducing FOXP3⁺ regulatory T cells (Figure 8C). This indicates a shift toward a more immunostimulatory landscape. Collectively, these *in vivo* findings corroborate our *in vitro* results, demonstrating that melatonin exerts its antitumor effects by targeting both tumor cell-intrinsic signaling and host immune responses.

## Discussion

This study provides a novel mechanistic rationale for the use of melatonin in TNBC, demonstrating that it simultaneously suppresses tumor progression and enhances immunosurveillance by inhibiting the FAK-PD-L1 signaling axis. TNBC is a highly aggressive subtype characterized by the absence of ER, PR, and HER2, rendering it unresponsive to endocrine and HER2-targeted therapies. Although recent advances have introduced novel targeted agents, cytotoxic chemotherapy remains the therapeutic mainstay. However, its efficacy is frequently undermined by adverse drug reactions (ADRs) and the development of drug resistance, contributing to poor clinical outcomes 30, 31. These challenges underscore the urgent need for adjunctive strategies to enhance therapeutic efficacy while minimizing toxicity in TNBC.

Melatonin, a circadian-regulated indoleamine, has gained attention in cancer biology for its multifaceted activities. Beyond regulating circadian rhythms, melatonin modulates key oncogenic pathways relevant to hormone-dependent malignancies 32, and epidemiologic studies have linked reduced melatonin levels to increased breast cancer risk 33. Consistent with reports of reduced melatonin secretion in cancer patients 34-36, our study revealed significantly lower serum melatonin levels in patients with TNBC compared to healthy controls, suggesting a potential correlation between melatonin deficiency and TNBC pathogenesis. Functionally, we demonstrated that melatonin exerts direct tumor-suppressive effects by inhibiting proliferation, colony formation, and tumorsphere growth *in vitro*, as well as suppressing tumor growth *in vivo*. Furthermore, melatonin exhibited potent anti-metastatic properties by reducing cell migration and invasion, addressing the aggressive dissemination characteristic of TNBC. Clinically, while cisplatin is a cornerstone of TNBC treatment, its utility is limited by systemic toxicity 37. Our findings indicate that melatonin potentiates cisplatin-induced cytotoxicity (Figure 6), suggesting a potent interaction that could allow for dose reduction and improved tolerability.

Beyond its direct cytotoxic effects, melatonin is increasingly recognized as an immunomodulator. Immune evasion in cancer is often facilitated by the upregulation of checkpoint proteins such as PD-L1, which suppresses T cell activation 38. In this study, melatonin suppressed both basal and IFN-γ-induced PD-L1 expression in TNBC cells. In our *in vivo* model, this was accompanied by increased infiltration of CD3⁺ and CD8⁺ T cells and a reduction in FOXP3⁺ regulatory T cells, indicating a shift toward an immunostimulatory tumor microenvironment. Given that current clinical strategies targeting the PD-1/PD-L1 axis predominantly rely on monoclonal antibodies, which can be limited by tissue penetrance and immune-related adverse events, our findings indicated the potential of melatonin as another modulator of PD-L1. Therefore, melatonin not only downregulates a key immune checkpoint but also promotes an antitumor immune response in TNBC. These findings support melatonin as a potential adjunct strategy to modulate PD-L1 and enhance antitumor immunity.

Mechanistically, our data suggested FAK as a critical target of melatonin in TNBC. FAK is implicated in proliferation, survival, and therapeutic resistance 39-43. Here, we observed that TNBC cells with low basal FAK expression were more sensitive to melatonin, and treatment led to dose-dependent reductions in FAK activity. Functional assays confirmed that genetic or pharmacological inhibition of FAK sensitized cells to melatonin, whereas FAK overexpression conferred resistance, establishing FAK as a key determinant of melatonin responsiveness. In addition, previous studies suggest that FAK is a regulator of immune evasion via PD-L1 upregulation 25-29, 42. Our observation that FAK overexpression attenuated melatonin-induced PD-L1 suppression supports the conclusion that the FAK-PD-L1 axis is a critical mediator of melatonin's immunomodulatory effects.

Regarding the downstream signaling, we observed that melatonin significantly reduced NF-κB expression in TNBC cells. Given that NF-κB is a canonical transcriptional regulator of PD-L1 44, these data suggest that melatonin-mediated PD-L1 suppression may involve NF-κB-associated transcriptional programs. Mechanistically, our results identify FAK as a key upstream regulator of PD-L1, and melatonin treatment resulted in the concurrent downregulation of FAK/p-FAK, NF-κB, and PD-L1. These findings point to a coherent signaling axis in which melatonin inhibits PD-L1 primarily via FAK suppression, potentially by attenuating FAK-dependent NF-κB signaling 28. However, as we did not directly assess NF-κB transcriptional activity or perform specific rescue experiments, the precise causal contribution of NF-κB to FAK-mediated PD-L1 regulation in this context remains to be fully characterized and warrants further investigation.

Finally, regarding clinical translatability, the dosage of 50 mg/kg used in this murine model was based on previous studies in murine models 19, 45. To address the translational relevance, we calculated the Human Equivalent Dose using the FDA-recommended body surface area normalization method 46, which corresponds to approximately 4.05 mg/kg for humans. While this dosage exceeds physiological levels, melatonin is distinguished by its high therapeutic index. Clinical studies and systematic reviews have demonstrated that doses in this range are well-tolerated in humans with minimal adverse effects 47. Therefore, the dosage employed in this study is within a feasible range for clinical investigation as a high-dose adjuvant therapy.

## Conclusion

In summary, this study provides compelling evidence that melatonin exerts multifaceted antitumor effects in TNBC. It directly inhibits the proliferation, migration, and survival of TNBC cells while simultaneously enhancing cisplatin cytotoxicity and favorably modulating the immune response. Mechanistically, these effects are mediated, at least in part, through the downregulation of the FAK-PD-L1 signaling pathway. Melatonin's ability to enhance T cell infiltration and reduce regulatory T cells within the tumor microenvironment further underscores its potential as an immunomodulatory agent. Given its favorable safety profile, accessibility, and pleiotropic mechanisms of action, melatonin represents a promising adjunctive therapeutic strategy for TNBC. Future clinical investigations are warranted to validate its efficacy and optimize its integration into existing treatment regimens.

## Data Availability

The data that support the findings of this study are available from the corresponding author upon reasonable request.

## Abbreviations

ER: estrogen receptor
PR: progesterone receptor
HER2: human epidermal growth factor receptor 2
TNBC: triple-negative breast cancer
EMT: epithelial-mesenchymal transition
FAK: focal adhesion kinase
PD-L1: programmed death-ligand 1
siRNA: small interfering RNA
